# Exercise-induced release of cardiac and skeletal muscle injury biomarkers in patients with chronic myeloid leukemia receiving TKI therapy

**DOI:** 10.1038/s41408-023-00861-3

**Published:** 2023-05-30

**Authors:** Lando Janssen, Neeltje A. E. Allard, Vincent L. Aengevaeren, Thijs M. H. Eijsvogels, Silvie Timmers, Nicole M. A. Blijlevens, Maria T. E. Hopman

**Affiliations:** 1 Radboud Institute for Health Sciences, Department of Hematology, Radboud University Medical Center, Nijmegen, The Netherlands; 2 Radboud Institute for Health Sciences, Department of Physiology, Radboud University Medical Center, Nijmegen, The Netherlands; 3 Human and Animal Physiology, Wageningen University, Wageningen, The Netherlands

**Keywords:** Chronic myeloid leukaemia, Signs and symptoms

Dear Editor,

Patients with chronic myeloid leukemia (CML) often require lifelong treatment with tyrosine kinase inhibitors (TKIs). Although TKI treatment results in an excellent prognosis, adverse events are frequently reported and may differ between first- and second-generation TKIs. Imatinib has been associated with a higher risk of skeletal muscle complaints compared to nilotinib, while cardiovascular events are more frequently reported with nilotinib [1]. Physical activity is a non-pharmaceutical strategy to enhance both muscle function and reduce cardiovascular disease risk [2], and hence could be of added significance in patients receiving TKI treatment. However, physical activity may provoke or exacerbate muscle complaints, which is supported by our observation that patients who experience adverse events on TKIs are less physically active [3]. It is unclear if exercise acutely increases cardiac or skeletal muscle damage in TKI users and whether this differs between TKIs.

To assess and compare exercise-induced cardiac- and skeletal-muscle injury, we measured high-sensitivity cardiac Troponin I (hs-cTnI) and creatine kinase (CK) levels before and after exercise in an age- and sex-matched group of 14 CML patients receiving nilotinib, 14 CML patients receiving imatinib, and 14 non-CML controls (57% males, 53 ± 11 years old). Patient characteristics and study methods have been described in more detail elsewhere [4]. Importantly, TKI treatment duration did not differ between nilotinib users (32 months (IQR: 13–69)) and imatinib users (49 months (IQR: 28–157); *P* = 0.16) and fat free mass index did not differ across groups (19.2 ± 3.1 kg/m^2^, 19.3 ± 1.7 kg/m^2^, and 19.1 ± 2.9 kg/m^2^ in nilotinib, imatinib, and control subjects, respectively; *P* = 0.98). One (7%) patient in the nilotinib group had a history of myocardial infarction, compared to none of the participants in the imatinib or control group. Antihypertensive medication was used by three (21%) nilotinib-treated CML patients and two (14%) CML patients receiving imatinib treatment, while none of the controls received antihypertensive drugs. Four (29%) nilotinib users were on statin treatment during study participation. One patient in the imatinib group dropped out of the study due to a treatment switch to dasatinib before exercise testing. Blood samples were collected at baseline (pre-exercise), directly after 1 h of submaximal cycling exercise (at resting heart rate + 70% of heart rate reserve) and 2 h post-exercise. hs-cTnI concentrations were analyzed using the Alinity i STAT High Sensitive Troponin-I assay (Abbott Laboratories, Abbott Park, IL, USA) with an established URL of 15.6 ng/L in females and 34.2 ng/L in males according to the manufacturer’s protocol. The reference values for CK levels are 170 U/L and 190 U/L for females and males, respectively, according to the manufacturer’s protocol (Roche Cobas). Biomarkers were logistically transformed and compared across groups using one-way analysis of variance due to skewed distribution. Biomarker concentrations over time were analyzed using linear mixed models.

Baseline hs-cTnI levels were significantly higher in CML patients receiving imatinib treatment (5.0 ng/L [IQR 2.6–16.5]) compared to non-CML controls (2.1 ng/L [IQR 1.5–2.4]; *P* = 0.002), while nilotinib users showed intermediate levels (3.0 ng/L [IQR 2.3–4.6], Fig. 1). Hs-cTnI levels increased following exercise (2.6 ng/L [IQR 2.1–5.5] at baseline to 4.3 [IQR 3.1–7.2] directly post-exercise to 4.1 ng/L [IQR 2.7–7.0] 2 h post-exercise; *P* = 0.03) for the total group. The magnitude of the increase was not different across groups (*P* = 0.39). At baseline two (15%) patients in the imatinib group had hs-cTnI levels exceeding 99th percentile (upper reference limit), whilst all participants in the nilotinib and control group had hs-cTnI levels below 99th percentile. One (7%) nilotinib-treated patient and two (15%) imatinib-treated patients had hs-cTnI levels exceeding the 99th percentile post-exercise.

**Fig. 1:**
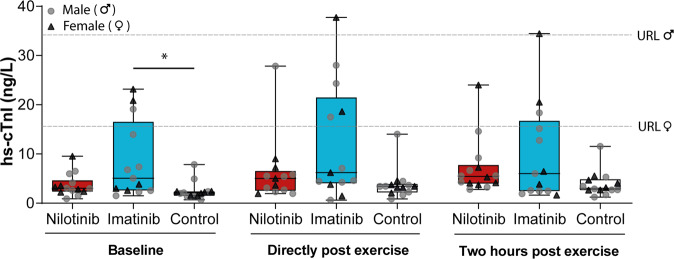
High-sensitivity cardiac Troponin I (hs-cTnI) concentrations across groups at baseline, directly post-exercise and 2 h post-exercise. Symbols represent individual values. Boxes represent interquartile (25th to 75th percentile) ranges, whiskers represent minimal and maximal values, and the horizontal line indicates median. Directly post-exercise, data of one patient in the nilotinib group was missing due to technical difficulties. Abbreviations: hs-cTnI high-sensitivity cardiac Troponin I; URL upper reference limit.

Baseline CK levels were significantly higher in imatinib-treated CML patients (211 U/L [IQR 138–284]) compared to non-CML controls (96 U/L [IQR 57–137]; *P* = 0.009, Fig. 2). The CK levels of nilotinib users (125 U/L [IQR 80–175]) were not different from the other groups. CK levels did not increase post-exercise; (135 U/L [IQR 84–182] at baseline to 126 U/L [IQR 89–204] directly post-exercise to 128 U/L [IQR 96–213] 2 h post-exercise; *P* = 0.46), and this response was comparable across groups (*P* = 0.98). At baseline, elevated CK levels were observed in two (15%), seven (58%), and one (7%) participants in the nilotinib, imatinib, and control groups, respectively, which increased to three (21%) and nine participants (69%) post-exercise, in nilotinib and imatinib, respectively. One (7%) participant in the control group had elevated CK levels at all time points.

**Fig. 2:**
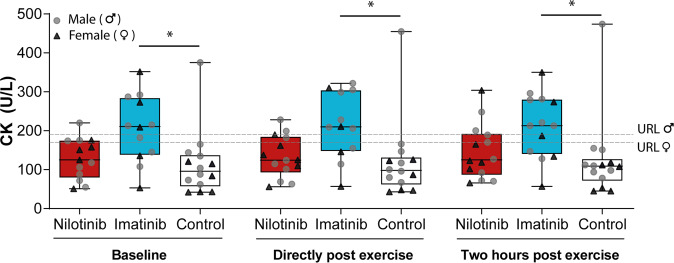
Creatine kinase (CK) concentrations across groups at baseline, directly post-exercise and 2 h post-exercise. Symbols represent individual values. Boxes represent interquartile (25th to 75th percentile) ranges, whiskers represent minimal and maximal values, and the horizontal line indicates median. Data of one patient in the nilotinib group and one patient in the imatinib group was missing at baseline, and data of one imatinib-treated patient was missing directly after exercise due to technical difficulties. hs-cTnI high-sensitivity cardiac Troponin I, URL upper reference limit.

The significantly higher hs-cTnI levels at baseline in imatinib users may have clinical relevance since higher resting hs-cTnI concentrations have been associated with increased mortality and cardiovascular disease morbidity in both the general and patient populations, even if below the upper reference limit [5, 6]. Additionally, post-exercise hs-cTnI concentrations may reveal myocardial vulnerability as the exercise-induced hs-cTnI elevations above the 99th percentile has been shown to be an independent predictor for mortality and cardiovascular events in a group of middle-aged and older long-distance walkers [7]. Cardiovascular events have been predominantly associated with nilotinib therapy [1], but the potential cardiotoxicity of imatinib treatment is subject of debate. In case reports, imatinib therapy has been associated with severe heart failure of unknown etiology [8]. However, in two following retrospective studies, both including ~1000 patients, the prevalence of imatinib-induced congestive heart failure was only 0.2–2% [9, 10], making cardiotoxicity of imatinib less likely. In this study, we found that patients receiving imatinib therapy had higher resting hs-cTnI concentrations compared to nilotinib and controls, with two patients exceeding to upper reference limit at baseline. Hs-cTnI concentrations were not correlated with TKI treatment duration (*r*_*s*_ = 0.13, *P* = 0.53), and did not associate with the history of cardiovascular disease or the use of antihypertensive medication. Together, this may suggest increased chronic myocardial damage in our imatinib patient group when compared to the nilotinib-treated CML patients and non-CML controls, although results should be interpreted cautiously due to the relatively small sample size. Imatinib-induced cardiotoxicity is poorly understood, but mitochondrial dysfunction as well as superoxide production have been proposed as underlying mechanisms [8, 11]. Following exercise, concentrations increased similarly in all groups, thus making increased TKI-induced myocardial vulnerability to exercise less likely. However, it should be noted that exercise-induced troponin release is highly related to the product of both the intensity and duration of an exercise stimulus [12]. Although our exercise stimulus might have been too moderate to reflect maximal exercise-induced troponin release, our exercise regimen was selected to be feasible for all our study participants.

Elevated CK levels are more common in imatinib-treated CML patients when compared to patients receiving nilotinib and non-CML controls. In fact, 58% of the patients receiving imatinib therapy had elevated CK levels at baseline, although levels were only slightly increased (well below 10 times URL). These elevated CK levels in imatinib-users have been observed in other studies [13]. We previously found no correlations between the imatinib-induced increased CK levels and self-reported muscle complaints [14]. CK levels did not significantly increase following exercise in our study. This may suggest that 1 h of submaximal cycling exercise did not exacerbate skeletal muscle damage in patients receiving TKI treatment. Increased CK values have been found directly post-exercise in both healthy individuals and patients [15], but it should be noted that it may take several days until CK levels reach peak values after exercise, depending on the level of training as well as type, intensity and duration of exercise [16].

Collectively, CML patients receiving nilotinib did not show increased cardiac or skeletal muscle injury biomarkers at rest or post-exercise in this exploratory study. CML patients receiving imatinib showed higher levels of hs-cTnI and CK at rest compared to non-CML controls, suggesting that imatinib may potentiate chronic cardiac and skeletal muscle injury. However, the exercise-induced release of both cardiac- and skeletal-muscle biomarkers was not augmented by either TKI, suggesting that both imatinib and nilotinib users can perform exercise without increased risk of exercise-induced cardiac and skeletal muscle injury. This may have important clinical implications as physical activity is known to reduce cardiovascular risk, which is of special importance to nilotinib users but may also be of significance for imatinib users as some of these patients showed signs of chronic myocardial injury in our study. Data of our study indicate that TKI use should not be a limiting factor for CML patients to engage in physical activity.

## Data Availability

Data are available from the authors upon reasonable request.
